# A Novel Method for Reducing the Effect of Tonic Muscle Activity on the Gamma Band of the Scalp EEG

**DOI:** 10.1007/s10548-012-0255-9

**Published:** 2012-09-11

**Authors:** Judith F. Nottage, Paul D. Morrison, Steve C. R. Williams, Dominic H. ffytche

**Affiliations:** 1 Department of Neuroimaging, Institute of Psychiatry, King’s College London, London, UK; 2 The Biomedical Research Centre, Institute of Psychiatry, King’s College London, London, UK; 3 Department of Old Age Psychiatry, Institute of Psychiatry, King’s College London, London, UK

**Keywords:** EEG, Gamma, Artefact, EMG, Motor

## Abstract

Neural oscillations in the gamma band are of increasing interest, but separating them from myogenic electrical activity has proved difficult. A novel algorithm has been developed to reduce the effect of tonic scalp and neck muscle activity on the gamma band of the EEG. This uses mathematical modelling to fit individual muscle spikes and then subtracts them from the data. The method was applied to the detection of motor associated gamma in two separate groups of eight subjects using different sampling rates. A reproducible increase in high gamma (65–85 Hz) magnitude occurred immediately after the motor action in the left central area (*p* = 0.02 and *p* = 0.0002 for the two cohorts with individually optimized algorithm parameters, compared to *p* = 0.03 and *p* = 0.16 before correction). Whilst the magnitude of this event-related gamma synchronisation was not reduced by the application of the EMG reduction algorithm, the baseline left central gamma magnitude was significantly reduced by an average of 23 % with a faster sampling rate (*p* < 0.05). In comparison, at left and right temporo-parietal locations the gamma amplitude was reduced by 60 and 54 % respectively (*p* < 0.05). The reduction of EMG contamination by fitting and subtraction of individual spikes shows promise as a method of improving the signal to noise ratio of high frequency neural oscillations in scalp EEG.

## Introduction

It has become increasingly evident from animal studies that frequencies above 20 Hz play a key role in the active functioning of the brain. Furthermore interest has grown into gamma band changes in various pathological states, such as psychosis (Green et al. 1999; Haig et al. 2000; Uhlhaas et al. 2006; Williams et al. 2009; Woo et al. 2010). Before 2007 many EEG groups assumed that the gamma band of the EEG could be extracted reliably using traditional analysis methods. However, two key publications have since challenged this assumption. The first was in 2007 by Whitham et al. (2007), who showed that the major part of the EEG gamma signal disappears with temporary muscle paralysis. The second blow was the demonstration by Yuval-Greenberg et al. (2008), in the following year, that most of the widely reported induced broad-band gamma peak at 200–300 ms after a visual stimulus does not originate in the brain (Yuval-Greenberg et al. 2008; Keren et al. 2010), which followed concerns raised by Trujillo et al. (2005), a few years earlier, about possible saccade related gamma band artefacts. Instead the primary source of the gamma peak is the extra-ocular muscles of the eye during micro-saccades, and its timing corresponds to the rebound in micro-saccades after the initial post-stimulus inhibition of saccades. This saccade associated artefact has been found to even contaminate intra-cranial recordings, (Jerbi et al. 2009; Kovach et al. 2011) and large surface EMG bursts can also sometimes be detected in intra-cranial recordings (Otsubo et al. 2008), but to a much lower degree than the surface EEG.

The conclusion from these two key papers, namely that most of the EEG gamma band was artefactual and not neuronal in origin, has presented a major challenge to ongoing gamma band research (Michel and Murray 2009). The methods presented here and in a previous paper which addresses the saccade associated artefact (Nottage 2010), were developed out of the necessity to determine whether meaningful results could be obtained in studies to explore EEG gamma changes in, for example, psychiatric disorders of cognition and psychosis.

Existing methods for dealing with scalp EMG consist of either source separation methods, such as Independent Component Analysis (ICA) or Canonical Correlation Analysis (Delorme et al. 2007; McMenamin et al. 2010; Shackman et al. 2010), or alternatively regression in the frequency domain, using high frequency power as a regressor (McMenamin et al. 2009). The first method is only suited to extracting high amplitude EMG that is volume conducted to multiple channels, such as the contraction of the Masseter muscle during jaw clenching, or the mouth and tongue muscles during speech (De Vos et al. 2010). However, in the scalp there are many small muscle fibres which are tonically active if there is even a small amount of scalp tension. Each of these muscles represents a separate source of electrical artefact, often detectable at only one or two electrode locations. Freeman and colleagues showed that the spatial frequency of EMG from these sources is similar to EEG, so that spatial filtering cannot be used (Freeman et al. 2003). This implies that spatial source separation is not a viable method for correcting for these artefacts. In fact there is controversy about the effectiveness of ICA techniques for removing EMG effects even in the alpha frequency band (Shackman et al. 2009; McMenamin et al. 2010; Olbrich et al. 2011) and ICA is inadequate for the gamma band (Shackman et al. 2010). The second method, since it uses the high frequency gamma power as a regressor, is only suitable for lower frequencies and cannot be used to retrieve the gamma signal.

The most important methodological development presented in this paper is a novel approach to reducing the effect of scalp and neck EMG. This artefact reduction method arose from the observation that scalp and neck muscle spikes had specific waveforms in the time domain which allowed them to be distinguished from the EEG (Goncharova et al. 2003). This gave rise to the possibility of generating computer algorithms which could reduce the former without interfering with the latter.

In order to determine whether the algorithms described here can help in the extraction of neuronal gamma, we needed to examine their effects on an established gamma signal. Intra-cranial recordings show that an increase in high gamma power is associated with self-paced motor tasks in contra-lateral motor and somatosensory areas (Pfurtscheller and Neuper 1992; Crone et al. 1998; Ball et al. 2008), although there may be a much smaller gamma activation ipsilaterally. This gamma peak is also observable using MEG (Cheyne et al. 2008) and can, under certain conditions, sometimes be detected in the EEG (Ball et al. 2008; Demandt et al. 2012). The high gamma occurs predominantly just after the commencement of the movement as well as at its termination. However, with a rapid motor act, such as a button press, the gamma at the start and end merge into one peak occurring just after the movement. Crone et al. (1998) reported that high gamma event related synchronisation (ERS) typically began “within 100 ms of the motor response onset”. Ball and colleagues found that the high gamma was greatest in the 60–90 Hz frequency band but also spread to higher frequencies (Ball et al. 2008), whereas Cheyne and colleagues used MEG and found a peak at 65–80 Hz which was maximal between 100 and 250 ms (Cheyne et al. 2008). Thus, previous studies indicate that the magnitude of neuronal motor/somatosensory gamma, in the 65–85 Hz band, is increased in the 250 ms period after a button press. This gamma signal was used in the present study to test the effectiveness of the EMG reduction algorithm.

## Materials and Methods

### Subjects

The EEG data used was from two independent groups, of eight subjects each, collected using two different EEG recording protocols. Both groups performed a self-paced motor task. There were five females and three males in group 1 and an equal number of male and female, right handed subjects in group 2.

### Self-paced Motor Task

During EEG recording, subjects were instructed to press a button with their index finger, on a game-pad located next to their right hand, approximately once per second. After 70 button presses “Task Finished” appeared on the computer screen. The oral instructions given to the subjects differed between the two cohorts in that, for the first group only, subjects were instructed to press the button approximately once every second. In the second group this constraint was dropped, so that the subjects had greater freedom in choosing when to press the button.

### EEG Recording

The EEG was recorded with Neuroscan 4.3 software and a 64 channel cap using the 10–20 system. Two electrodes were attached close to the outer canthi of the left and right eyes to record the left and right Saccade Muscle Potential (SMP) signals. A linked mastoid reference was used for the EEG recording for group 1 and a linked earlobe reference for group 2. Fast sampling rates were used, of 2,000 Hz for the first group, with a resolution of 0.1 μV, and 5,000 Hz, with a resolution of 0.02 μV for the second group. The filter settings at recording were 200 and 1,000 Hz respectively for the low pass filter and 0.05 Hz for the high pass filter. The epoched data was exported to MATLAB for further analysis.

### Methods for Dealing with Artefacts

#### Method for Reduction of Power-line Noise

The artefacts caused by power-line noise have the advantage that they have simple waveforms consisting of a 50 Hz (or 60 Hz in other countries) sine wave combined with harmonics. A noise cancellation method was used as described previously (Nottage 2010). In this procedure the fundamental sine waves are extracted by band pass filtering the recorded power-line noise. This is phase shifted by π/2 to obtain the cosine corresponding to the original sine wave. An improved method for obtaining the harmonic signals is to use the basic trigonometric relationship in Eq. 1, which was applied in this analysis. Linear regression is used to find the weights for the original, phase shifted and harmonic waveforms in each electrode. The noise signals multiplied by the weights are finally subtracted from the raw EEG.

1 $$ \,{ \sin }\left( {{\text{A}}\;{ + }\;{\text{B}}} \right)\;{ = }\;{ \sin }\;{\text{A}}\;{ \cos }\;{\text{B}}\;{ + }\;{ \cos }\;{\text{A}}\;{ \sin }\;{\text{B}} $$

#### Method for Reduction of the Saccade Muscle Potential

The correction method used is described in detail previously (Nottage 2010). It involves recording the SMP artefact at the corners of the left and right eyes, followed by regression and subtraction of a weighted average of these potentials from the EEG signals.

#### Method for Reduction of Spike Muscle Artefacts

**Fig. 1:**
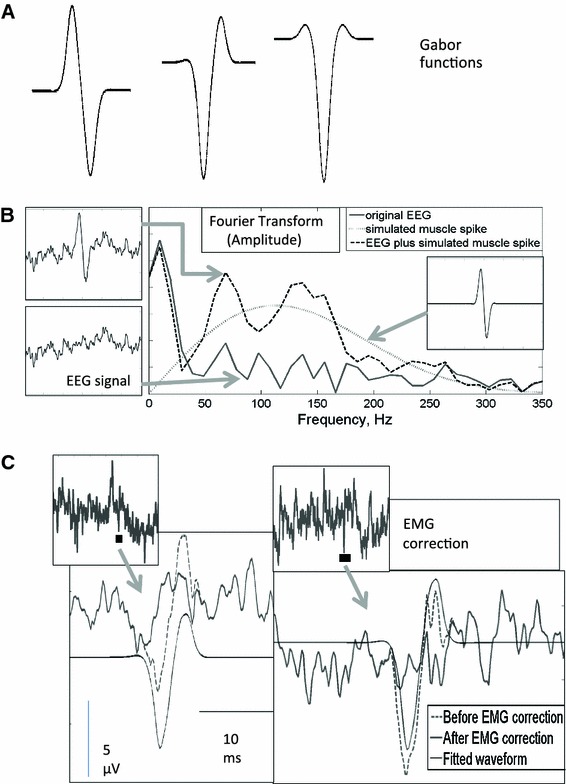
Mathematical modelling of scalp muscle spikes. **a** Three examples of the Gabor functions used with three different phase shifts between the Gaussian and the sine-wave. **b** A simulated muscle spike, shown on the *inset plot* on the large graph, was added to a sample of EEG with minimal EMG contamination. The amplitude was found using a fast Fourier transform (410 ms window). The* plot* on the *bottom left* shows the original EEG, and the combined EEG and the Gabor waveform is shown above it. **c** Two examples of fitting and subtraction of EMG spikes from the EEG. The *dotted line* shows the original EEG, the *light solid line* is the fitted Gabor waveform and the *dark solid line* is the corrected EEG signal. The *insets* above show the uncorrected EEG on a longer time-scale, with the *black boxes* indicating the location of the particular muscle spike

The basis of this method is that muscle spikes have specific waveforms, which can be readily modelled mathematically using Gabor functions. A Gabor function consists of a sine-wave modulated by a Gaussian. Different muscle spike waveforms are modelled by changing the phase of the sine wave with respect to the Gaussian. Three examples of these functions with different phases of sine waves are shown in Fig. 1a, together with an example of the effect on the frequency spectrum when a simulated muscle spike is added to a sample of EEG with low EMG contamination.

It was observed, by inspection of the EEG, that muscle spikes, presumably arising from a train of action potentials in the scalp or neck muscles, always show a sharp potential change over a time period of 3.5 ms. However, not all large voltage changes over 3.5 ms are due to muscle spikes; the EEG will only be corrected where the algorithm can fit a matching Gabor function. The first stage in the muscle reduction algorithm is the detection of time points where there is a sharp change in electric potential over 3.5 ms. These points are taken as the first estimates of the point of zero crossing of the sine-wave. In order to find the mid-point of the Gaussian a feature of the first and third differential of the Gabor function is used. Near the centre of the Gaussian the function approximates to a sine-wave, and differentiating multiple times makes this feature more pronounced. Differentiating a sine wave twice produces the same wave, but scaled and inverted. Consequently at the centre of the Gaussian the first and third differential become inversely correlated with each other. After identifying the point with the highest gradient, as above, a search is made, within the nearest few milliseconds, for the point where the negative correlation between the first and third differential is at its greatest. This is used to generate the first guess at the centre of the Gaussian. The spike was ignored if the correlation coefficient was lower in magnitude (less negative) than a fixed threshold (−0.78 for group 1 and −0.84 for group 2). For the initial detection step, only, we used EEG signals which had been high pass filtered with a finite impulse response (FIR) 10 Hz filter in order to reduce the influence of large, slow frequency changes.

**Fig. 2:**
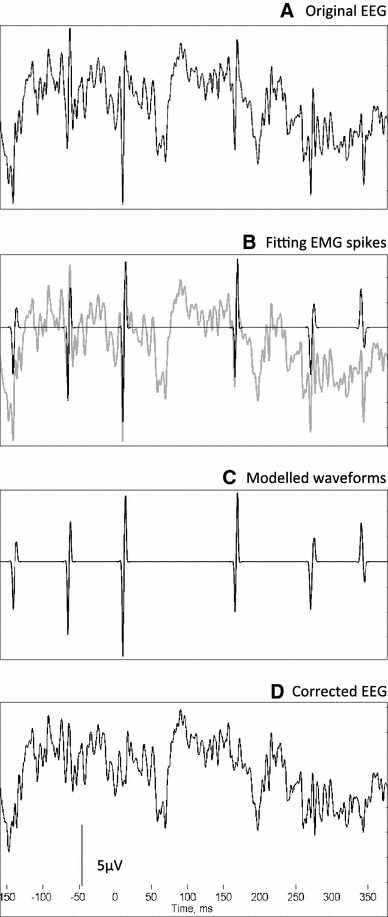
EMG correction: The correction of a segment of EEG from a *left central* location (C3–CZ). **a** The original EEG, which contains *sharp, spiky waveforms* indicative of scalp EMG. **b** Mathematical modelling of the spikes. **c** The fitted Gabor waveforms to be subtracted. **d** The corrected EEG, containing *fewer sharp, spiky waveforms*

The other parameters of the function are then estimated. The time parameter for the spread of the exponential is estimated by calculating the ratio of the gradient at the centre point of the function to the gradient 1 ms previously. The expected gradient ratios are calculated for a range of time spread parameters and used to estimate the time spread parameter for the muscle spike in question. Next an iterative process is used to improve on the first guess of the centre time of the function. This is done by shifting the time by 0.01 ms and testing if the correlation between the Gabor function and the EEG has increased or decreased. Finally the amplitude of the spike is found by regression of the Gabor waveform and the EEG, and this waveform is subtracted from the EEG. With the 5,000 Hz sampling rate it was necessary to include extra, higher frequency, Gabor functions in the model, in order to capture higher frequency components of the muscle spikes. The modelled waveform is then subtracted from the EEG signal, as shown in Figs. 1 and 2.

Changing key threshold values, in particular the minimum voltage change in 3.5 ms which is considered by the algorithm, can affect its performance. This means that there is scope for optimizing the algorithm on the basis of both the appearance of the corrected EEG trace and the prominence of the motor related gamma peak. However, selecting individual parameters which give the best gamma peak could artificially inflate the significance of this peak. This can be avoided by using identical, standard, parameters. Therefore, the artefact correction was carried out with both standard and optimised thresholds. The standard threshold used for the 3.5 ms change was 7 μV, whilst the optimised ones ranged from 4–9 μV.

### Time–Frequency Analysis

Sliding epochs, consisting of suitable numbers of points for carrying out a fast Fourier transform, were cut from the EEG at 5 ms intervals and multiplied by a Hanning widow in MATLAB. For group 1, which had a 2,000 Hz sampling rate, the epoch length was 256 ms (512 points), whilst for group 2, using a 5,000 Hz sampling rate, the epoch length was 409.6 ms (2,048 points). Fast Fourier transforms were carried out to obtain the amplitude values. The amplitudes from a left central electrode dipole (C3–CZ) and a right central electrode dipole (C4–CZ) were used to give left and right central gamma magnitudes. Left and right temporo-parietal dipoles (TP7–CPZ, TP8–CPZ) were included for comparison.

One complicating issue is that of rejection of artefactual epochs. It is standard practice within the EEG field to delete periods of EEG with gross artefacts, and in the case of muscle artefacts the magnitude at high frequencies is often used to trigger rejection. For this study any button press epoch for which the 60–130 Hz amplitude exceeded a threshold was rejected. However, as there was a substantial variation in 60–130 Hz magnitude across subjects, using the same low threshold led to a rejection of all epochs from some subjects, whilst, with a high threshold, there was a failure to reject clear EMG bursts in other subjects. The aim of the rejection procedure is to improve the data by rejecting the most contaminated epochs, whilst retaining sufficient data for further analysis, and therefore using a single fixed threshold was unacceptable. Instead the threshold was determined, for each subject, using Eq. 2. The parameters *a* and *b* were set such as to avoid rejecting more than 30 of the 70 trials, whilst ensuring all obvious muscle bursts were rejected. The maximum threshold allowed was an average of 0.4 μV/Hz over the whole 60–130 Hz frequency band.

2 $$ \,{\text{Amplitude}}\,{\text{threshold}}\;{ = }\;{{a}}\; \times \;{\text{average}}\, 6 0\,{\text{to}}\, 1 3 0\,{\text{Hz}}\,{\text{amplitude}}\,{\text{over}}\,{\text{all}}\,{\text{trials}}\;{ + }\;{{b}} $$

where* a* = 1.5 and* b* = 0.06 μV/Hz.

The epochs with above threshold 60–130 Hz amplitude in the post SMP reduction but pre EMG reduction data were selected for deletion. After EMG artefact reduction the 60–130 Hz activity associated with large muscle bursts becomes less apparent. However, in order to make a fair assessment of the effect of the algorithm it is important that these same high 60–130 Hz epochs are rejected when calculating the amplitude values in other the conditions, such as with EMG correction. Therefore, the selected epochs were also deleted when calculating the amplitude values before SMP correction, and after EMG correction. As a final step, any trial in which the maximum high gamma magnitude was more than six standard deviations above the mean was considered to be anomalous and was also rejected.

### Statistical Analysis

#### Detection of High Frequency Gamma Associated with the Motor Task

The amplitudes following the motor action were compared with the baseline period of 1 s before the button press. However, some button presses were closer than one second apart, which would cause overlapping of the baseline period with previous gamma activations. In this case the whole of the available time, starting 250 ms after the previous button press, was used as the baseline period. For each subject the mean peak and baseline high gamma amplitudes were calculated. Statistical significance was tested using a paired, one tailed *t* test, across subjects but independently for the two cohorts. This process was repeated for ipsilateral (right) central high gamma magnitude. In the event of group-wise significant effects being observed, it would still be of interest to know whether a significant high gamma peak would be detectable on a within subject basis. Therefore, the above test was repeated for each subject. This was done by calculating the gamma amplitude in the 65–85 Hz frequency band in the 250 ms after the time of the button press, for each trial, compared to the baseline gamma for that trial. A paired, one tailed *t* test was applied for each subject. We also carried out some supplementary analysis on frequencies up to 130 Hz, to see if broader band activity was detectable in the EEG.

#### Effect of Artefact Correction Algorithms

The difference in 65–85 Hz gamma power in the 750 ms before the button press was calculated for each subject before artefact correction, after SMP correction and after both SMP and EMG correction. The significance of reductions in gamma with artefact correction was tested, across subjects, for each cohort separately, using one-tailed *t* tests. Significance was tested first for the SMP correction and then for the subsequent EMG correction. Also the effect of each algorithm on the magnitude of the baseline corrected motor gamma peak was tested for each cohort, but using a two tailed *t* test since it was unclear whether this peak would increase or decrease in amplitude.

To assess the topographic distribution of the gamma ERS, the nearest midline electrodes were used for re-referencing the EEG before subsequent EMG reduction. Z scores for each of multiple electrode locations, and for each subject, were calculated as the mean baseline corrected ERS, divided by the standard deviation. These Z scores were averaged across subjects and plotted.

## Results

### Behavioral Data

In group 1 the mean time between button presses was 1.53 s, with 0.40 s standard deviation between subjects. Group 2 tended to have longer times between button presses than group 1, with a mean of 1.91 s as well as a larger standard deviation between subjects of 1.14 s.

### Effect of Correction of the Extra-ocular Muscle (SMP) Artefact

In both cohorts there was a strong trend towards a very small reduction in the mean, over all time-points, of the left and right central 65–85 Hz amplitude after the SMP artefact reduction process (group 1: 0.14 % = 2.6 nV, *p* = 0.057; group 2: 0.2 % = 3.0 nV, *p* = 0.076). Neither group showed a significant difference between the magnitudes of the baseline corrected, left central, gamma ERS before and after SMP reduction.

### Reduction in Amplitude and Power with the Application of the Algorithm

The amplitude of the 65–85 Hz band, in the 750 ms prior to the button press, was significantly reduced in C3–CZ in group 2 by the optimized EMG artefact correction (23 % reduction = 0.34 μV, *p* < 0.05). In group 1 there was a strong trend towards a reduction (16 % reduction = 0.29 μV, *p* = 0.067). Also there were larger, statistically significant reductions (60 % reduction = 2.28 μV, *p* < 0.05, 54 % reduction = 1.89 μV, *p* < 0.05) in the temporo-parietal electrodes, TP7–CPZ and TP8–CPZ, in the group 2 analysis. The reduction at the right central location did not quite reach significance (47 %, 1.1 μV, *p* = 0.06). There was no significant difference between the EMG reduction in the right and left locations for either of these pairs (*p* = 0.27 for C3–CZ/C4–CZ, *p* = 0.44 for TP7–CPZ/TP8–CPZ).

### Is Neuronal Motor/Somatosensory Gamma Detectable in the Scalp EEG?

**Fig. 3:**
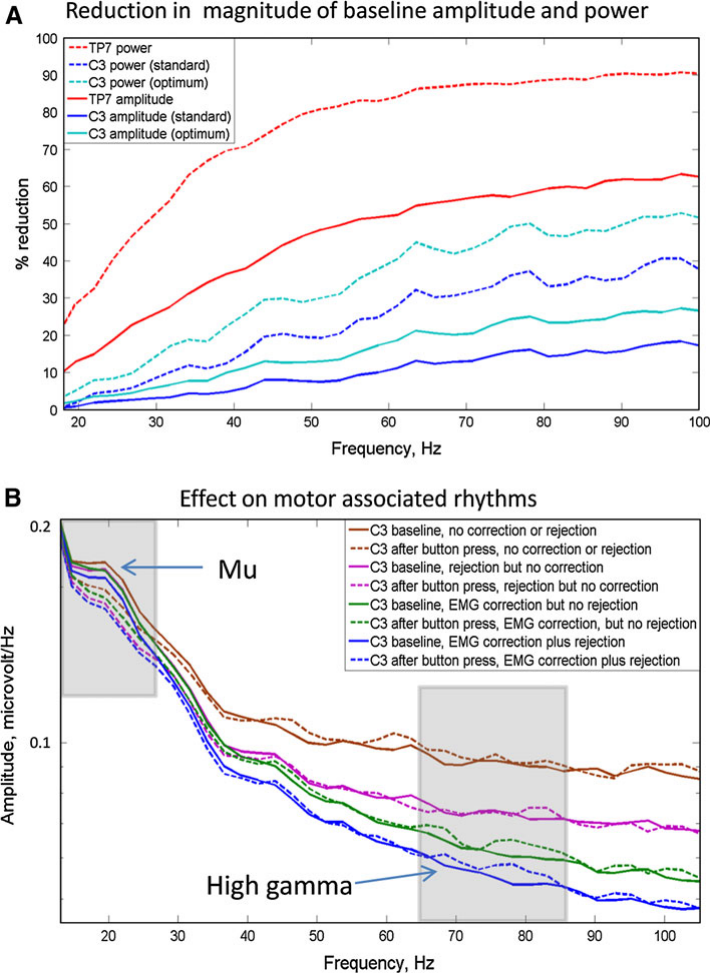
Amplitude changes and EMG reduction. This shows the effect of EMG artefact reduction on the EEG in group 2. **a** Percentage reduction of amplitude and power at different stages of EMG reduction in TP7 and C3. Note that power values are considerably larger than equivalent amplitude values. **b** Mean spectra at baseline (750 ms before the button press*, solid lines*) and after button press (0–250 ms, *dotted lines*). Below 22 Hz the effect of EMG is much less than the effect of neural Mu rhythm changes. Above 28 Hz the EMG contribution becomes larger than any event related changes in neural oscillations. Note that using this optimized EEG correction, the 65–85 Hz gamma ERS is relatively unaffected by the inclusion (*green lines*) or exclusion (*blue lines*) of epochs containing large muscle bursts (Color figure online)

**Fig. 4:**
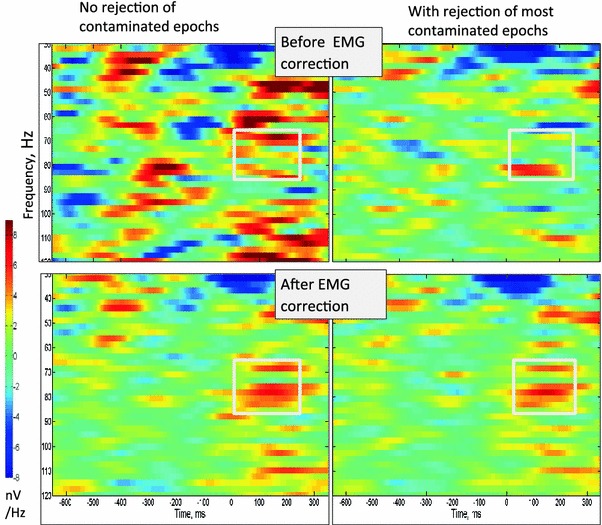
Mean time frequency plots for group 2: these are averaged across all eight subjects, showing the effect of rejection of the most contaminated epochs and the application of the EMG reduction algorithm. The *white box* shows the time and frequency used for statistical testing. The mean amplitude in the 750 ms before the button press was subtracted from the signal. Note that the motor gamma peak is visible after either rejection of contaminated epochs or EMG reduction with or without rejection. However, without EMG correction the baseline at 65–85 Hz is 23 % higher and the motor peak does not reach significance

**Fig. 5:**
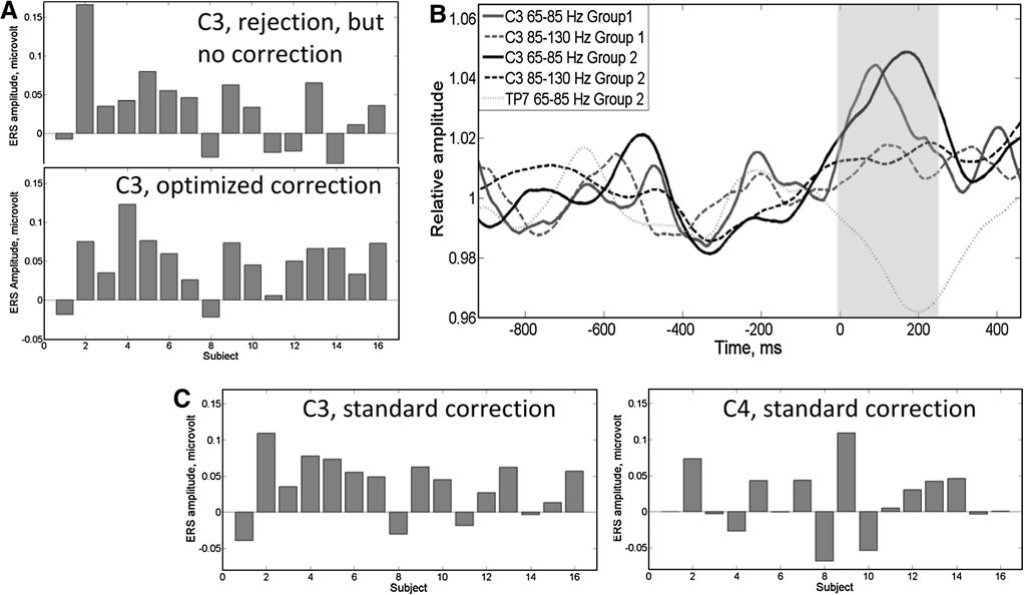
Event related change in 65–85 Hz gamma after button press. **a** This shows the gamma amplitude change, as a percentage of baseline, for all subjects from both studies. After EMG correction the left motor gamma (C3–CZ) becomes more consistent across subjects. **b** The amplitude changes at 65–85 Hz, relative to the previous 750 ms, are shown for the two separate cohorts. There is a similar, event-related increase in gamma, in both groups, which is not visible at TP7 (group2), and is much less pronounced in the higher, 85–130 Hz band. Note that as the sampling frequency differed between studies the window length for the Fourier transform also differed, and therefore the temporal resolution was not the same. This is why the trace is smoother in group 2. **c** The gamma ERS is left lateralised as it is not significant on the right side at C4–CZ. Identical analysis parameters were used for both electrode locations

Comparing across subjects there was a highly significant peak in left central gamma immediately after the button press in each of the two cohorts as can be seen in Figs. 3b, 4 and 5. For group 1 *p* = 0.03 both before and after EMG reduction with standard parameters, and *p* = 0.02 with individually optimized parameters. For group 2 *p* = 0.014 (65–85 Hz) and *p* = 0.011 with a broader frequency band (65–130 Hz), after scalp EMG reduction with standard parameters and, *p* = 0.0002 with optimized parameters (65–85 Hz), but the peak was not statistically significant before EMG reduction for group 2 (*p* = 0.16). In neither cohort was there a significant right central gamma peak immediately after the motor action (*p* = 0.12 at 65–85 Hz, *p* = 0.14 at 65–130 Hz in group 2). With group 2, after EMG correction using standard parameters, there was no significant event related change at the right temporo-parietal location (TP8–CPZ, *p* = 0.34), although at the left temporo-parietal electrodes (TP7–CPZ) there was a trend towards a reduction in gamma amplitude after the button press, (*p* = 0.08, two-tailed *t* test) as shown in Fig. 5. The event related changes in gamma on the left and right including all 16 subjects are shown in Fig. 5, together with the left percentage changes before EMG correction.

**Fig. 6:**
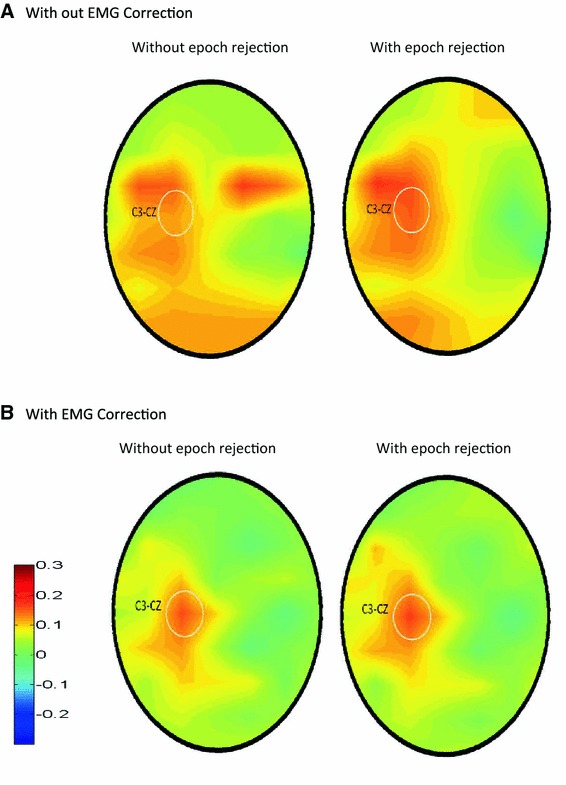
Topographical distribution of gamma ERS. Topographic plots of the mean Z score across all 16 subjects for the 65–85 Hz gamma ERS. Increased scalp and neck *EMG* can be seen in some posterior and front-temporal electrodes before correction, but after correction activity is limited to the left central area. The* plots* are derived from 42 bipolar derivations with one electrode of each pair located on the *midline*

Also within subject comparisons using the corrected EEG gave a significant left central gamma peak (*p* < 0.05) in six of the 16 subjects, as shown in Table 1. In only one of the 16 subjects was the peak significant on the right hand side. The topographic distribution of the mean within subject Z scores, averaged across all 16 subjects, is shown in Fig. 6b. It can be seen that the 65–85 Hz ERS has a left central distribution, centred on C3–CZ. However, there also appears to be increased neck and Temporalis muscle activity around the button press time, which is observable prior to EMG correction in Fig. 6a.

**Table 1:** Within subject significance levels of the 65–85 Hz ERS at C3–CZ

	1	2	3	4	5	6	7	8
Group 1: subject								
*p* value before EMG correction, with rejection	>0.5	0.35	**<0.01**	0.07	0.21	0.07	0.28	>0.5
*p* value after EMG correction, with rejection	>0.5	0.35	**<0.01**	0.07	**0.01**	0.08	0.24	>0.5
Group 2: subject								
*p* value before EMG correction, with rejection	**0.02**	0.15	>0.5	>0.5	0.08	>0.5	0.37	0.2
*p* value after EMG correction, with rejection	**<0.01**	0.08	0.39	0.12	**<0.05**	**0.01**	0.13	**0.02**

*Note* The same epochs were rejected from the data before and after EMG reduction. The *p* values <0.05 are shown in bold type

### Is the Neuronal Gamma Left Intact?

If the neuronal gamma were reduced by the algorithm then the event-related gamma peak would also be reduced. However, in neither group was there a significant reduction in this peak, even using standard algorithm parameters. On the contrary, in group 2 there was a significant increase in the magnitude of the baseline corrected peak using optimized parameters (0.015 μV before, 0.052 μV after, *p* = 0.023: two tailed *t* test). Also, as shown in Table 1, whilst only two, out of 16 subjects, showed a significant 65–85 Hz ERS (*p* < 0.05) prior to EMG correction, six had a significant ERS after EMG correction. Also, of the remaining ten subjects, five showed a trend towards a gamma ERS (*p* < 0.15), after artefact reduction.

## Discussion

### The Gamma Band of the Scalp EEG

We have demonstrated that an event related motor gamma band peak can be reliably detected with as few as eight subjects and a protocol lasting under 2 min. However, although the gamma peak can be reliably detected on a group-wise basis with only 70 button presses, this is insufficient to allow detection of significant gamma peaks on all individual subjects. Therefore, an increased number of button presses is to be recommended for studies aiming to detect gamma in individual subjects.

The extent of EMG contamination, without correction, can be seen from the spectra in Fig. 3a. In agreement with changes in the central EEG under paralysis (Whitham et al. 2007, 2008), EMG dominates the uncorrected signal down to about 28 Hz, and the true brain oscillations only become the largest component of the EEG below 22 Hz. However, it remains to be seen whether this applies to brain areas other than the central regions studied here.

### Effectiveness of Scalp EMG Artefact Reduction

#### Can the Algorithm Reduce the Effect of EMG?

Whitham’s group reported 33 % (CZ) to 93 % (T8) reduction in gamma power with muscle paralysis (Whitham et al. 2008). However, they used an average reference and retained the most artefactual peripheral channels in their average. It is likely that most of the 33 % contamination reported at CZ originated from the average reference used. Since the scalp and neck EMG magnitude is lower at electrode locations near the top and centre on the head, EMG will be lower if, instead of using an average reference, a midline channel, such as CZ, is used as a reference. Also the EMG reduction algorithm used in this paper works optimally if there is only one source of EMG spikes to model. Introducing EMG from a second group of muscle fibres, via a reference electrode, produces coincident spikes which cannot be readily fitted. Another important factor is that the values in the current study are expressed in amplitude changes rather than power changes. Since power is the square of the amplitude, power increases due to EMG will always be larger than amplitude changes: a power reduction of 33 % corresponds to an amplitude reduction of 18 % and a power reduction of 93 % corresponds to an amplitude reduction of 73 % (see Fig. 3a). The fact that a 23 % mean reduction was obtained in high gamma amplitude at C3–CZ means that a substantial proportion of the effect of the scalp EMG at these electrode locations has been removed. Nevertheless, despite this large reduction in noise, muscle spikes were still sometimes observed in the corrected EEG trace, meaning that there is further room for improvement in the efficacy of the algorithm.

It should also be remembered that even if all artefactual contamination were removed from the gamma band of the scalp EEG it would still consist of at least three residual signals: neuronal gamma oscillations, harmonics from lower frequencies and the gamma component of the broad-band EEG signal. The mean event related increases in the 65–85 Hz band were 0.045 and 0.052 μV for the two groups, which is less than one-sixth of the 0.29 and 0.34 μV reductions in amplitude with EMG correction, and less than one twentieth of the residual 1.54 and 1.12 μV. Whilst we would contend that the event-related changes and harmonics of lower frequencies originate in the brain, it is less clear what proportion of the large residual broadband signal has a neuronal origin.

#### Does the Algorithm Also Reduce Neuronal Gamma?

Although the baseline gamma may well contain some level of residual EMG contamination, the residual gamma ERS is similar to that observed intra-cranially, so it is reasonable to make the assumption that this is neuronal in origin. In group 2, particularly, the statistical significance of the event related increase was greater after EMG reduction than before. Also the amplitude of the task related gamma was not significantly reduced by the algorithm in either of the groups. To the contrary, in group 2 a significant increase in the event-related gamma could be detected. This is probably due to the reduction in the gamma magnitude after the motor act being less than the reduction at baseline, because of removal of destructive interference between the EMG waveforms and motor gamma.

#### Does the Algorithm Add Any Correction Errors into the Data?

It should be remembered that the algorithm only subtracts modelled muscle spike waveforms from the data. If spike waveforms were incorrectly subtracted, for example where there was no actual muscle spike in the EEG, the effect would be the equivalent of increasing EMG activity, by adding muscle spikes to the signal, and would lead to an increase in the overall magnitude of gamma. However, we did not find increases in gamma magnitude for within mean subject values at baseline.

### Future Work

To our knowledge this represents the first attempt to use fitting of individual muscle spikes to deal with EMG artefact, and it is clear that this is a promising strategy. The value of the algorithm lies in its ability to distinguish between the waveforms of presumed neuronal EEG and surface EMG. However, it should be pointed out that there are limitations, since sometimes neuronal EEG may contain waveforms that approximate to EMG spikes and vice versa. Therefore, one line of future work will be to attempt to quantify the algorithm’s ability to separate the two waveforms, and also to explore additional approaches for distinguishing neuronal EEG and surface EMG. In view of the fact that this is a first generation algorithm of its kind, it is very likely that future generations of the algorithm will give significant further improvement in performance. The faster sampling rate of 5,000 Hz appeared to be preferable to 2,000 Hz, since it gave a larger and more statistically significant reduction in the EMG noise. This is probably due to the fact that the increase in temporal resolution allows for a more accurate fitting of the muscle spike waveforms. However, it is unclear whether even higher sampling rates could make sufficient additional improvement to out-weigh the increased data storage requirements.

The EMG reduction method represents another step in a pipeline to reduce non-neuronal noise from the gamma band of the EEG, following on from the reduction of the power-line and micro-saccade associated steps. Unfortunately at least one potential hurdle remains before the prize of a clear neuronal gamma signal is obtained, namely harmonics from lower frequency waveforms. EEG waveforms in the 8–20 Hz band tend to be non-sinusoidal, and are frequently saw-tooth in shape. Such waves produce harmonics at whole number multiples of their frequencies, which tend to have the greatest effect in the lower gamma bands, and can show event-related changes. Thus, the detection of a low gamma ERS may be confounded by ERD of Mu and beta rhythms with sharp peaks. An important future direction for research is to develop methods which will distinguish between these harmonics and the smaller, genuine gamma waves. In the mean-time it will be necessary either to focus on the higher gamma frequencies, or else on a brain region or task that does not generate large alpha waves.

Finally it should be noted that, using this novel EMG reduction algorithm, the high frequency gamma peak was visible without rejecting any segments of EEG data contaminated by large bursts of EMG activity. Previous EEG studies have concluded that scalp EMG contamination prevents the high gamma increase being a useful control signal for brain–computer interface BCI applications (Williams et al. 2009). Should improvements in the speed of the algorithm be made in the future, it may prove possible to use the gamma band in on-line or BCI applications without false alarms caused by muscle bursts.

## Conclusions

It has been shown that an increase in the amplitude of 65–85 Hz gamma can reliably be detected during the 250 ms following a button press over contra-lateral central areas in the surface EEG. Our novel EMG reduction algorithm can significantly reduce the magnitude of the scalp EMG without reducing the size of the gamma peak. Especially with a very fast sampling rate the signal to noise ratio of the gamma is increased, as demonstrated by increased significance of the gamma peak. We find that the method of using mathematical modelling to reduce scalp EMG helps us to detect the gamma signal in our EEG.
